# Prescribing of Electronic Activity Monitors in Cardiometabolic Diseases: Qualitative Interview-Based Study

**DOI:** 10.2196/jmir.8107

**Published:** 2017-09-23

**Authors:** Alice Bellicha, Sandrine Macé, Jean-Michel Oppert

**Affiliations:** ^1^ Institute of Cardiometabolism and Nutrition University Pierre et Marie Curie-Paris 6 Paris France; ^2^ Laboratory of Bioengineering, Tissues and Neuroplasticity University Paris-Est Créteil France; ^3^ Department of Marketing Ecole Supérieure de Commerce de Paris-Europe Paris France; ^4^ Assistance Publique-Hôpitaux de Paris Department of Nutrition Pitie-Salpetriere University Hospital Paris France

**Keywords:** cardiometabolic diseases, physical activity, physicians’ perspectives, prescriptions, mobile health, telemedicine, mHealth, electronic activity monitors, fitness tracker, accelerometer, smart pedometer

## Abstract

**Background:**

The prevalence of noncommunicable diseases, including those such as type 2 diabetes, obesity, dyslipidemia, and hypertension, so-called cardiometabolic diseases, is high and is increasing worldwide. Strong evidence supports the role of physical activity in management of these diseases. There is general consensus that mHealth technology, including electronic activity monitors, can potentially increase physical activity in patients, but their use in clinical settings remains limited. Practitioners’ requirements when prescribing electronic activity monitors have been poorly described.

**Objective:**

The aims of this qualitative study were (1) to explore how specialist physicians prescribe electronic activity monitors to patients presenting with cardiometabolic conditions, and (2) to better understand their motivation for and barriers to prescribing such monitors.

**Methods:**

We conducted qualitative semistructured interviews in March to May 2016 with 11 senior physicians from a public university hospital in France with expertise in management of cardiometabolic diseases (type 1 and type 2 diabetes, obesity, hypertension, and dyslipidemia). Interviews lasted 45 to 60 minutes and were audiotaped, transcribed verbatim, and analyzed using directed content analysis. We report our findings following the Consolidated Criteria for Reporting Qualitative Research (COREQ) checklist.

**Results:**

Most physicians we interviewed had never prescribed electronic activity monitors, whereas they frequently prescribed blood glucose or blood pressure self-monitoring devices. Reasons for nonprescription included lack of interest in the data collected, lack of evidence for data accuracy, concern about work overload possibly resulting from automatic data transfer, and risk of patients becoming addicted to data. Physicians expected future marketing of easy-to-use monitors that will accurately measure physical activity duration and intensity and provide understandable motivating feedback.

**Conclusions:**

Features of electronic activity monitors, although popular among the general public, do not meet the needs of physicians. In-depth understanding of physicians’ expectations is a first step toward designing technologies that can be widely used in clinical settings and facilitate physical activity prescription. Physicians should have a role, along with key health care stakeholders—patients, researchers, information technology firms, the public, and private payers—in developing the most effective methods for integrating activity monitors into patient care.

## Introduction

Physical inactivity is recognized as a leading cause of noncommunicable diseases, including cardiometabolic conditions such as type 2 diabetes, obesity, dyslipidemia, and hypertension [1,2]. Despite the well-established benefits of physical activity for preventive care and management of these diseases, it remains underprescribed by physicians [3]. The rapid expansion of mobile technology, including electronic activity monitors (EAMs), presents an opportunity for encouraging physicians to prescribe physical activity. EAMs typically track daily movement, mainly the number of steps taken, with sensors both recording acceleration and providing feedback to the user via a monitor display or a smartphone app [4]. Two recent randomized controlled trials showed that EAMs associated with individually tailored feedback may increase physical activity in individuals with overweight and obesity or type 2 diabetes [5,6]. However, a mismatch between information technology firms that are developing new technologies and the physicians who use them has been reported [7]. In addition, knowledge about the needs of physicians when prescribing EAMs to patients is very limited. The aims of this qualitative study were therefore (1) to explore how specialist physicians prescribe EAMs to patients presenting with cardiometabolic conditions, and (2) to better understand physicians’ motivations for and barriers to prescribing such monitors.

## Methods

### Procedures and Participants

We conducted qualitative semistructured interviews to investigate the prescribing of EAMs in patient care, following guidelines from the Consolidated Criteria for Reporting Qualitative Research (COREQ) [8]. This type of analysis is used when prior research on a subject exists but is incomplete or could benefit from further description [9].

Participants were hospital physicians with expertise in management of patients with cardiometabolic diseases (ie, type 1 and type 2 diabetes, obesity, dyslipidemia, and hypertension). Recruitment took place from March-May 2016 in one public university hospital in Paris (Assistance Publique-Hôpitaux de Paris, France). We contacted 11 physicians whom we knew by email and personally invited them to participate. All agreed to participate. We used purposive sampling to achieve a varied composition in terms of their sex, experience, status, and fields of expertise (Table 1).

**Table 1:** Characteristics of physicians interviewed.

Code	Sex	Age range (years)	Medical specialty	Status
P1	Male	50-59	Obesity	Hospital physician, university professor
P2	Female	30-39	Obesity, diabetes	Hospital physician
P3	Female	50-59	Cardiology, obesity	Hospital physician, private practice
P4	Male	≥60	Obesity, diabetes	Hospital physician, university professor
P5	Male	≥60	Diabetes	Hospital physician, university professor
P6	Female	50-59	Diabetes	Hospital physician
P7	Female	40-49	Diabetes	Hospital physician, university professor
P8	Male	50-59	Endocrinology, dyslipidemia	Hospital physician, university professor
P9	Male	50-59	Endocrinology, dyslipidemia	Hospital physician, university professor
P10	Male	50-59	Hypertension	Hospital physician, university professor
P11	Female	50-59	Obesity	Hospital physician, private practice

Diversity within the sample of specialist physicians was important so as to take into account different opinions and further improve understanding. Among the physicians, 4 were heads of their departments at the time of the study and 1 had headed a department in the recent past. Participants received a verbal explanation on the aim of the study prior to the interviews. The study complied with standards set by the Declaration of Helsinki, and we obtained written consent from all participants.

### Interviews

We conducted all interviews at work in the physicians’ offices. No one was present besides the participant and the researcher conducting the interviews. Interviews lasted 45 to 60 minutes and were carried out by the first author (AB), a PhD student in sports science who had attended a training course on qualitative research prior to the study. We developed a discussion guide, including open-ended questions, prior to the first interview. We asked physicians to explain how they handled the issue of physical activity with their patients. As such, we examined 4 stages of physical activity counselling: (1) initial assessment of physical activity, (2) prescription, (3) patient education, and (4) follow-up evaluation [10]. Next, we asked physicians to describe their experiences with EAMs in routine clinical care, motivations for and barriers to prescription, and their expectations. All interviews were audiotaped with the consent of participants and transcribed verbatim. Field notes were made during the interviews to facilitate data analysis and interpretation [8].

### Data Analysis

We used thematic analysis via a directed approach [9]. After completion of the interviews, repeated reading of transcripts enabled familiarity with the data. Then, 1 investigator coded the transcripts according to predefined categories (initial evaluation, prescription, education, and follow-up evaluation). Data that could not be coded using the initial categories were identified and later analyzed to determine whether they represented a new category. We discussed the coding process with each other throughout the analysis. Physicians’ comments are presented verbatim (translated from French into English) in the Results and are identified by the physicians’ code (Table 1).

## Results

### Prescribing of Physical Activity

Interviewed physicians consistently recommended physical activity to their patients, mainly orally. Several barriers to prescribing physical activity emerged.

#### Lack of Evaluation Tools

Prescribing of physical activity was seen as requiring a specific diagnosis, often not available to physicians; they all reported difficulties in accurately assessing physical activity, especially its intensity.

Patients are not aware of their physical activity. They think they perform some physical activity just because they walk. But in reality, that’s not physical activity. I want them to sweat.

P10

#### Opportunity Cost

Prescribing physical activity was described as a difficult, time-consuming task, especially when compared with prescribing a drug. Discussing physical activity with rather reluctant patients and reinforcing their motivation for physical activity would take more time and require greater involvement.

It’s much more difficult to give advice on moving 30 minutes a day than to say “Take this pill.”

P2

In addition, accompanying patients in their daily practice of physical activity, although of prime importance, is not a main task for physicians. They deplored the lack of available solutions for patient follow-up.

Apart from pedometers, we have nothing to propose. There is no physical activity instructor in my department, and this is lacking.

P7

#### Perceived Risk

Prescribing physical activity was considered more risky than prescribing drugs, mainly because physical activity cannot generally be accepted as a cure compared with drug treatment, the symbolic nature of which was mentioned.

You can provide a solution in less than 5 minutes with a drug prescription, just by saying “Try this and you’ll get better.” It’s like selling dreams, a cure, in just one sentence, it’s extraordinary. With physical activity, you can’t do that, unless maybe you find tools that tell you how to prescribe it.

P10

Also, loss of credibility possibly resulting from lack of efficacy of physical activity appeared to be a barrier to prescription.

When we say to patients “Your blood glucose level will go down” and this does not turn out to be the case, we lose credibility. Whereas with medication, we know it’s going to work.

P8

### Prescribing of Electronic Activity Monitors

#### Experience With Electronic Activity Monitors

Physicians agreed that an increasing number of patients own an EAM.

Many patients have already downloaded an app for tracking physical activity.

P11

Most physicians said that they spent time analyzing data with patients.

I ask where they stand, if it helps, I encourage them, look at the results and comment on them.

P7

But only 4 physicians had already recommended either an EAM or the pedometer included in mobile phones, and only on rare occasions.

#### Perceived Benefits

Physicians acknowledged that EAMs might help patients to assess their physical activity level and might potentially motivate them, providing novelty and a recreational aspect in the context of long-term management of chronic disease. Some physicians also described EAMs as a means of improving the patient-doctor relationship.

It enables us to discuss something concrete.

P2

#### Lack of Clinical Utility

Barriers to prescription of EAMs included lack of evidence on data validity and reliability, and lack of interest in data collected. Physicians felt that data collected by EAMs are unsuitable for meeting the goals they define for the patient.

I can’t see the benefit of an electronic activity monitor for me. The information isn’t useful.

P10

Patients who bring their connected data, I have no time for that. Their number of steps...it’s not a goal I’ve defined.

P10

Physicians expected an accurate measurement of relevant outcome (eg, physical activity duration and intensity, time spent sitting) that would be presented in the form of summary scores readily understandable by both the patient and physician. They also wished to personalize criteria such as physical activity goal, type of data synthesized, period of analysis, and prompts and feedback sent to the patient.

Positive feedback, okay, but not all the time.

P4

Some physicians also suggested that physical activity data should be translated into motivating benefits, such as long-term health improvement (life-years gained), better short-term disease management (lower blood glucose, lower insulin dose, etc), and improved well-being.

#### Learning and Searching Cost

Physicians reported a lack of knowledge of available devices and difficulty in keeping aware of the continuously growing newly marketed devices.

I can’t be asked to learn about the available monitors. I don’t have time. It’s not the doctor’s role.

P8

Convenience, ease of use, and prior knowledge of EAMs were strongly awaited. Physicians also feared work overload that would result from automatic data transfer, that is, data that would be automatically transferred electronically to the physician between medical consultations.

The problem with connected devices is the additional workload. Patients send me emails all the time.

P2

In contrast, they did state that they wanted to discuss the data during the medical consultation.

The patient must bring data, otherwise I won’t have time to analyze them.

P1

#### Monitoring and Privacy Costs

Physicians pointed out the risk of patients becoming addicted to data.

The main problem with self-monitoring is the risk of addiction.

P3

The question is, how can I correctly use the electronic activity monitor and know I’m making progress, without becoming addicted to it?

P5

They also highlighted the risk of potential control of insurance coverage over the patients.

If physical activity data are going to be sent to private insurers for a bonus-malus contract, then the answer is no.

P5

#### Financial Issues

The high cost of devices was considered a barrier to their prescription. Most physicians recommended a selling price under €50 (about US $56) so as to be affordable for patients, and by analogy with the price of blood pressure self-monitoring devices. However, they explained that the monitors must have real added value to justify the expense.

Patients are not willing to pay high prices. The monitor must have real added value.

P6

Reimbursement by public or private insurance was not viewed as essential. Physicians believed that renting the EAMs for a limited time period rather than purchasing it could decrease costs and risk of addiction to data, and emphasize its educational role.

### Prescription of Self-Monitoring Devices

Blood pressure or blood glucose self-monitoring devices were frequently prescribed, with the principal aim of improving diagnosis and optimizing drug treatment.

Self-monitoring of blood glucose provides real information on whether the insulin dose is the right one.

P2

When devices that are connected were available, physicians recommended them because they seem easier to use in everyday life settings. However, they were reticent about automatic data transfer. One physician explained that, in the context of chronic diseases, automatic data transfer goes against the principle of patient autonomy.

Transmitting blood glucose to a nurse who will tell the patient what to do, it’s ridiculous, even more so in diabetes, that requires immediate action.

P5

## Discussion

### Principal Findings

Activity monitors are becoming increasingly popular among the general public. However, our results suggest that hospital physicians with expertise in management of patients with cardiometabolic diseases have not yet integrated EAMs into routine clinical care, which contrasts with their frequent prescription of other types of self-monitoring devices, such as blood pressure or blood glucose monitors.

While most physicians had never recommended EAMs, they acknowledged their potential to increase patient motivation through precise quantification of physical activity. However, they were concerned about data validity, which has been shown in several published studies to be insufficient [11]. EAMs are consumer-grade monitors often validated by the company only after market launching, without external validation [12].

The physicians we interviewed also questioned the clinical utility of recording step counts. Physicians followed current physical activity guidelines that recommend a given duration and intensity of physical activity but do not recommend a cutoff for steps taken per day [13-15]. Given the inability of patients to accurately estimate intensity, physicians expected this outcome to be measured by EAMs. It is surprising that companies have not yet designed EAMs measuring physical activity intensity, since it could be easily obtained via minute-by-minute analysis of step counts. Walking cadence (ie, number of steps/min) is recognized as a valuable reflection of intensity, and thresholds have been proposed to categorize intensity based on cadence [16]. Cadence has the advantage of being easily interpretable by patients and physicians. Real-time feedback of walking cadence would improve patients’ perception of intensity, highlighting the potential educational role of EAMs [17]. Moreover, by helping physicians to assess physical activity, EAMs could overcome a major difficulty in prescribing physical activity.

Beyond EAMs’ role in data collection, physicians attach great importance to the feedback provided by EAMs. They expected this to be easily understandable by both patient and physician, and to be presented in the form of summary scores over personalized periods of time. They also suggested that physical activity data be translated into short-term benefits related either to improved disease management (eg, better regulation of blood glucose, reduction in insulin dose) or to improved well-being. Emphasizing the benefits of physical activity would provide patients with immediate concrete rewards [18], which are known to have higher priority than greater but delayed rewards, and could therefore improve adherence to long-term lifestyle changes [19,20]. This proposition points out a major difference in how physicians use EAMs and blood glucose or blood pressure self-monitoring devices. The latter devices provide direct, more accurate and complete measures of health outcomes than does traditional monitoring. Their main benefits for physicians have been to optimize drug prescriptions and, for patients, to make appropriate treatment choices and motivate lifestyle changes [21]. Therefore, by analogy with widely adopted blood glucose and blood pressure monitors, EAMs could be designed to provide feedback concerning the benefits of physical activity for health outcomes. Such feedback would decrease the perceived risk associated with prescribing of physical activity. A simple translation of physical activity data into health benefits, as suggested by physicians, appears to be a feasible strategy that could be rapidly implemented by companies. Some authors have predicted that, in the near future, a single device will have the capacity to monitor a range of data, including both physical activity and relevant medical data [22], and will provide patients and physicians with a direct measure of physical activity benefits.

A striking finding of this study was that the learning and searching costs associated with use of EAMs prevented physicians from prescribing them. They deplored a lack of information about available EAMs and difficulties with choosing between the ever-growing number of devices on the market. In contrast, they appeared better informed about blood pressure or blood glucose monitors, which are regulated medical devices [23]. Lists of devices that have been independently validated for use in clinical practice are freely available [24], as are guidelines on how to use them for assessment and management of diabetes and hypertension, which is not yet the case for EAMs [21,25]. A broader adoption of EAMs will necessarily involve lowering learning and searching costs for physicians. Over the short term, the effort to gather information on the validity, features, or cost of commercially available EAMs could be assigned to other health care professionals, such as physical activity instructors. Over a longer term, EAMs that meet validity and effectiveness requirements of medical device regulations could be marketed, and the best practices to be shared between physicians and patients would be defined.

Physicians highlighted monitoring risks that patients might experience when using EAMs, especially that of addiction to data. Such risks have been described in patients using blood glucose and blood pressure self-monitoring devices [26,27]. Thus far, studies have suggested a decrease in adherence to EAMs over time, without mentioning the risk of addiction to physical activity data [22]. The physicians interviewed here supported the idea that patients should rent EAMs for a limited time period rather than purchasing them. Renting has the advantage of decreasing both the risk of addiction to data and financial costs that physicians consider too high to be affordable for patients of low socioeconomic status [28]. Physicians also warned against use of data by private insurers who might penalize insufficiently active patients. Such contracts have recently been authorized in some countries [29], although they are not authorized in others. This warning points to the critical issue of data privacy, now recognized as a priority by companies and regulators [29].

Finally, and surprisingly, all of the physicians we interviewed strongly opposed automatic data transfer, not only because of time constraints, but also because they considered that developing patient autonomy and self-care ability is a major aspect of patient education in the context of chronic diseases. The ability of wearable technology to transmit data to the physician is usually presented as attractive for clinical applications [30]. However, physicians have neither the time nor the desire to receive physical activity data, preferring to discuss data with the patient during the more traditional context of a medical consultation. Our data highlight the need for companies to work closely with physicians to determine when the contact with connected data is clinically useful, which may vary according to the disease, the type of data collected, and individual preferences [7].

### Strengths and Limitations

First, this qualitative study provides a perspective on attitudes of hospital physicians regarding EAMs and their integration into patient care. To our knowledge, this is the first study that specifically targeted hospital physicians with expertise in cardiometabolic diseases, a set of conditions with increasing prevalence worldwide. Hospital physicians are opinion leaders in the medical community and influence the prescription habits of their colleagues [31]. Second, we explored barriers to physical activity prescription, a necessary first step in understanding whether and how EAMs might encourage physicians to prescribe physical activity.

Our study has some limitations. First, most physicians interviewed were aged 50 years or older. Younger physicians may have different beliefs regarding EAMs, since adoption of new technologies is higher at younger ages [32]. Second, the relatively small number of participants was also a limitation, although it is acknowledged that the number of participants can be reduced when the degree of expertise increases [33]. Third, the physicians we interviewed were working in a university setting in Paris, France, and the findings may not directly apply to other medical settings around the world. Assessing the views of other profiles of physicians about EAM use would be useful. Fourth, we analyzed data using a directed content analysis, the main limitation being that researchers approach data with an informed and potential bias [9]. To limit bias, we asked only open-ended questions, so as to allow unexpected but relevant themes to emerge. In addition, we discussed coding of transcripts with each other throughout the analysis.

### Conclusions

The increased use of EAMs provides a timely opportunity to encourage prescribing of physical activity. EAMs have the potential to improve patient education and motivation through better assessment of physical activity, to enable a more precise prescription of physical activity, and to reinforce the patient-doctor relationship. However, hospital physicians with expertise in management of cardiometabolic diseases have not yet adopted EAMs. To do so, numerous barriers must be overcome. Important adaptations could be rapidly achieved (eg, measuring physical activity intensity through walking cadence, emphasizing health benefits), while others require more time and effort from key health care stakeholders (eg, defining best practices, regulating data privacy). This study pointed out questions related to the most effective use of EAMs for management of chronic diseases that should be explored in future studies.

## Abbreviations

COREQ: Consolidated Criteria for Reporting Qualitative Research
EAM: electronic activity monitor
